# Whole-Genome Sequence of Lactobacillus salivarius DJ-sa-01, Isolated from Chicken Small Intestine

**DOI:** 10.1128/MRA.00987-18

**Published:** 2018-10-11

**Authors:** Dongjun Kim, Mun-Ju Cho, Seungchan Cho, Yongjun Lee, Sung June Byun, Sukchan Lee

**Affiliations:** a Department of Genetic Engineering, Sungkyunkwan University, Jangan-gu, Suwon, Republic of Korea; b Animal Biotechnology Division, National Institute of Animal Science, Rural Development Administration, Wanju-gun, Jeollabuk-do, Republic of Korea; Georgia Institute of Technology

## Abstract

We have identified the whole-genome sequence of Lactobacillus salivarius DJ-sa-01, a potential probiotic strain for poultry, isolated from a chicken small intestine. We used the PacBio and Illumina platforms to obtain the sequence of the entire single circular chromosome.

## ANNOUNCEMENT

Immune-enhancing feed additives have been developed over recent years to replace antibiotics. Antibiotics are traditionally added to feed, in part to promote growth and control Salmonella-induced food poisoning and diarrhea in various species of farm animals (1). Probiotics, which modulate gut microbiota and confer health-promoting effects to the host, represent promising alternatives to antibiotics. Many studies are underway to develop probiotics for animal feed (2). Our ultimate goal was to isolate new promoter candidates that can express proteins (such as 3D8 scFv, which hydrolyzes viral nucleic acids) constitutively in Lactobacillus salivarius DJ-sa-01 (3).

Here, we present the whole-genome sequence of Lactobacillus salivarius DJ-sa-01, isolated from the small intestine of a 27-week-old female chicken. The study protocol and standard operating procedures were reviewed and approved by the Institutional Animal Care and Use Committee of the National Institute of Animal Science in Korea (NIAS2018273). Tissues from the small intestine were harvested and homogenized using 1.6-mm stainless steel beads. Bacterial isolates from homogenized tissues were grown using Man-Rogosa-Sharpe (MRS) agar, which is selective for Gram-positive bacteria. Cells were cultured and later maintained at −85°C using 80% glycerol. To extract genomic DNA, bacterial cell walls were first lysed with lysozyme and mutanolysin, followed by genomic DNA extraction using a G-spin genomic DNA extraction kit (iNtRON Biotechnology, Republic of Korea) (4). We performed a sequence comparison analysis of L. salivarius DJ-sa-01 and other L. salivarius strains reported in the NCBI database, specifically comparing 16S rRNA and DNA gyrase subunit B. We found that L. salivarius DJ-sa-01 16S rRNA had 99% sequence similarity and DNA gyrase subunit B had 98% sequence similarity to those of other L. salivarius strains.

For whole-genome sequencing, we used the PacBio RS II (Pacific Biosciences, USA) and Illumina HiSeq 2000 (Illumina, San Diego, CA, USA) platforms. Sequencing was performed at Macrogen (Seoul, Republic of Korea). First, paired-end libraries with an insert size of 252 bp were constructed for Illumina sequencing. As a result of 2 × 100-bp paired-end sequencing, 71,610,448 reads (100-bp read length) were generated. After long-read sequencing on the PacBio RS II platform, 1,262,204,072 bp were generated (141,529 reads, about 419-fold genome coverage). Raw sequence data were assembled by the Hierarchical Genome Assembly Process v3 (HGAP3) using SMRT Analysis software v2.3.0 (5). To compensate for any inaccuracies introduced by PacBio sequencing, error correction was conducted by making complete genome contigs with reads generated by Illumina sequencing. After hybrid assembly using the two combined sequencing methods, one contig was made, which is a circular chromosome harboring a size of 1,870,629 bp and GC content of 33.0%. By comparative genomic analysis, this constructed genome was 99% similar to that of L. salivarius UCC118. The chromosome included 1,703 putative coding sequences (CDS) annotated with Prodigal v2.6 software (6). Predictions for tRNA and rRNA genes were made using ARAGON v1.2 and RNAmmer v1.2 software, respectively (7). The genome of L. salivarius DJ-sa-01 comprises 78 tRNA genes and 22 rRNA genes.

### Data availability.

The chromosomal sequence of Lactobacillus salivarius DJ-sa-01 has been deposited at GenBank under the accession number CP029616.
